# Associations of Long-Term Exposure to PM_2.5_ and Its Constituents with Erythrocytosis and Thrombocytosis in Rural Populations

**DOI:** 10.3390/toxics11110885

**Published:** 2023-10-27

**Authors:** Yiquan Zheng, Yaling He, Ning Kang, Caiyun Zhang, Wei Liao, Yinghao Yuchi, Xiaotian Liu, Jian Hou, Zhenxing Mao, Wenqian Huo, Kai Zhang, Hezhong Tian, Hualiang Lin, Chongjian Wang

**Affiliations:** 1 Department of Epidemiology and Biostatistics, College of Public Health, Zhengzhou University, Zhengzhou 450001, China; 2 Department of Occupational and Environmental Health, Ministry of Education, Key Laboratory of Environment and Health, and State Key Laboratory of Environmental Health (Incubating), School of Public Health, Tongji Medical College, Huazhong University of Science and Technology, Wuhan 430074, China; 3 Department of Environmental Health Sciences, School of Public Health, University at Albany, State University of New York, Albany, NY 12222, USA; 4 State Key Joint Laboratory of Environmental Simulation & Pollution Control, School of Environment, Beijing Normal University, Beijing 100875, China; 5 Department of Epidemiology, School of Public Health, Sun Yat-sen University, Guangzhou 510275, China

**Keywords:** PM_2.5_, chemical constituents, erythrocytosis, thrombocytosis, rural

## Abstract

Evidence on the effect of long-term exposure to fine particulate matter (PM_2.5_) on erythrocytosis and thrombocytosis prevalence was limited. We aimed to investigate the association of PM_2.5_ and its constituents with the risks of erythrocytosis and thrombocytosis. The present study included a total of 33,585 participants from the Henan Rural Cohort at baseline between 2015 and 2017. A hybrid satellite-based model was employed to estimate the concentrations of PM_2.5_ mass and its constituents (including black carbon [BC], nitrate [NO_3_^−^], ammonium [NH_4_^+^], inorganic sulfate [SO_4_^2−^], organic matter [OM], and soil particles [SOIL]). The logistic regression model was used to assess the associations of single exposure to PM_2.5_ and its constituents with the risks of erythrocytosis and thrombocytosis, and the quantile G-computation method was applied to evaluate their joint exposure risk. For the independent association, the odds ratios for erythrocytosis/thrombocytosis with 1 μg/m^3^ increase was 1.049/1.043 for PM_2.5_ mass, 1.596/1.610 for BC, 1.410/1.231 for NH_4_^+^, 1.205/1.139 for NO_3_^−^, 1.221/1.359 for OM, 1.300/1.143 for SO_4_^2−^, and 1.197/1.313 for SOIL. Joint exposure to PM_2.5_ and its components was also positively associated with erythrocytosis and thrombocytosis. The estimated weight of NH_4_^+^ was found to be the largest for erythrocytosis, while OM had the largest weight for thrombocytosis. PM_2.5_ mass and its constituents were positively linked to prevalent erythrocytosis and thrombocytosis, both in single-exposure and joint-exposure models. Additionally, NH_4_^+^/OM was identified as a potentially responsible component for the association between PM_2.5_ and erythrocytosis/thrombocytosis.

## 1. Introduction

Erythrocytosis and thrombocytosis are medical conditions characterized by an abnormal increase in red blood cell count (RBC) or platelet count (PLT), respectively, beyond the specific normal medical reference range [1,2]. In clinical practice, erythrocytosis is diagnosed by an elevated concentration of hemoglobin (HGB) or hematocrit (HCT) [3]. Both erythrocytosis and thrombocytosis could be categorized as primary or secondary conditions. Primary erythrocytosis and thrombocytosis occur due to an autonomous increase in the production of RBCs or platelets, generally stemming from myeloproliferative disorders [2,4]. In contrast, secondary erythrocytosis and thrombocytosis are caused by increased levels of circulating erythropoietin or thrombopoietin in response to various factors, such as cancer, chronic inflammation, infection, as well as lung or heart disease [2,5]. Recent evidence has indicated that erythrocytosis was associated with high clonal hematopoiesis prevalence, as well as cardiovascular morbidity and mortality, and all-cause mortality [4]. Additionally, thrombocytosis has been linked to increased risk of cancer morbidity, cause-specific mortality, and all-cause mortality [6,7].

Fine particulate matter (PM_2.5_) is a major environmental concern that significantly contributes to the global burden of mortality and morbidity burden [8]. PM_2.5_ has the ability to penetrate the respiratory barrier and enter the bloodstream, thereby impacting hematological and immune functions through various pathways [9]. Previous studies have established a link between exposure to PM_2.5_ and diseases such as asthma, anemia, thrombosis, and other blood-related disorders [10,11,12]. However, there is a lack of investigations exploring the association of PM_2.5_ with erythrocytosis or thrombocytosis to date. Previous reports have examined the impact of PM_2.5_ on erythrocyte and platelet parameters, including RBC, HGB, HCT, and PLT. Nevertheless, the findings from these studies have been inconsistent and even contradictory [13,14,15,16]. These discrepancies may be attributed to differences in study design, population characteristics, and methods used for exposure assessment [13,17]. Additionally, the composition of PM_2.5_ mixtures is another significant contributing factor, as it is the primary determinant of overall impact [18,19]. Therefore, it is crucial to identify the specific components of PM_2.5_ that contribute to its detrimental effects on erythrocytosis and thrombocytosis. However, there is a lack of evidence regarding the effects of PM_2.5_ constituents on erythrocyte and platelet parameters. A previous cross-sectional study conducted among pregnant women during the third trimester observed inverse relationships of PM_2.5_, black carbon (BC), nitrate (NO_3_^−^), and organic matter (OM) with HGB levels [20]. Additionally, another study reported positive associations of PM_2.5_ with RBC and PLT; while no significant positive correlation was found between the constituents of PM_2.5_ and various erythrocyte parameters [21]. 

To our knowledge, there is currently insufficient evidence to determine the overall effects of PM_2.5_ and the specific toxic effect of PM_2.5_ constituents on erythrocytosis and thrombocytosis. Therefore, the purpose of the current study was to investigate the associations of long-term exposure to PM_2.5_ mass and its constituents with the risk of prevalent erythrocytosis and thrombocytosis and to identify the vital deleterious PM_2.5_ constituents.

## 2. Materials and Methods

### 2.1. Study Population

The study participants were drawn from the baseline of the Henan Rural cohort, which was previously described in detail [22]. Briefly, between 2015 and 2017, a total of 39,259 individuals aged 18 to 79 years were selected to participate in the baseline survey using multistage stratified cluster sampling from five districts in Henan Province, China. In the present study, we excluded 5287 subjects with missing relevant hematologic data, 326 subjects with severe medical conditions (including malignancies and kidney failure), and 61 pregnant women. Finally, 33,585 participants met the inclusion criteria. The Zhengzhou University Life Science Ethics Committee approved this study (reference code: [2015] MEC (S128)), which was conducted in accordance with the Declaration of Helsinki. Additionally, all participants provided informed consent prior to taking part.

### 2.2. Data Collection and Definition of Outcomes

Information on demographic characteristics, and lifestyle habits was collected using face-to-face questionnaires, while anthropometric data were obtained through measurement. In brief, marital status was categorized into two groups: married/cohabiting and unmarried/divorced/widowed. Education level was classified as primary school or below, junior high school, and senior high school or above, while per capita monthly income was categorized as <500 RMB, 500–999 RMB, and ≥1000 RMB. Moreover, smoking and drinking status were categorized as never, ever, and current. Among them, current smokers were defined as individuals smoking at least one cigarette per day for six sequential or cumulative months, and current drinkers were defined as those consuming alcohol at least 12 times per year. Physical activity was classified into three categories: low, moderate, and high. Furthermore, participants with a high-fat diet were defined as having an average daily intake of ≥75 g of poultry and/or livestock meat, while those with adequate vegetable and fruit intake were defined as having an average daily consumption of ≥ 500 g of vegetables and fruits [23]. Body mass index (BMI) was calculated according to the weight (kg) and height (m) of the individual.

Blood samples were obtained from all participants who had fasted for at least eight hours overnight and were detected by an automatic biochemical analyzer (XS-500i, SYSMEX, Tokyo, Japan) to derive hematological parameters (including HGB, HCT, PLT). Based on the 2016 criteria from the World Health Organization (WHO) guideline, erythrocytosis was defined as HGB concentration > 16.5 g/dL or HCT > 49% in men and HGB concentration > 16.0 g/dL or HCT > 48% in women [3]. According to local laboratory reference intervals and a previous study, thrombocytosis was defined as PLT > 400 × 10^9^/L [24].

### 2.3. Exposure Assessment

This study employed a hybrid model that combined output from satellite and ground observations with results from a chemical transport model simulation (GEOS-Chem; version 11–01; http://www.geos-chem.org, accessed on 20 March 2022). The model was used to estimate the concentrations of PM_2.5_ mass and its six constituents (BC, ammonium (NH_4_^+^), NO_3_^−^, OM, inorganic sulfate (SO_4_^2−^), and soil particles (SOIL)) at a resolution of 1 km. [25,26,27,28,29,30] In brief, the model estimated the monthly PM_2.5_ mass concentration by employing satellite-based aerosol optical depth (AOD) data and geographically weighted regression (GWR) at a spatial resolution of 1 km × 1 km, which was then calibrated using ground-based PM_2.5_ mass concentration data. [31] In addition, the GEOS-Chem CTM was used to evaluate the concentration of each PM_2.5_ constituent, by incorporating updated emission inventories and meteorological data obtained from a consistent reanalysis (MERRA-2). To determine the monthly exposure concentrations of PM_2.5_ mass and its constituents, this study used the geocoded home address and investigation date of participants with the GEOS-Chem CTM. The 3-year average concentrations, as a long-term exposure indicator in this study, were derived by aggregating the monthly concentrations before the participants were surveyed. The details regarding the simulation of GEOS-Chem are presented in Supplementary Materials.

### 2.4. Statistical Analysis

Frequencies (percentages) and means (standard deviations (SD)) were used to present statistical descriptions for categorical and continuous variables, respectively. Correspondently, differences between groups were compared for categorical variables using the Chi-square test and, for continuous variables, using Student’s *t*-test. Pearson correction analysis was used to evaluate the correlations among PM_2.5_ constituents, and the logistic regression model was utilized to evaluate their independent associations with erythrocytosis and thrombocytosis. Moreover, restricted cubic splines (with three knots at 25th, 50th, and 75th percentiles) were employed to investigate the dose–response associations of PM_2.5_ mass and its constituents with erythrocytosis and thrombocytosis. To specify more responsible constituents, the constituent-PM_2.5_ model and constituent residual model were further applied in addition to the single-pollutant model [32,33]. The constituent-PM_2.5_ model was utilized in order to control the potential confounding effects, by introducing PM_2.5_ mass as a surrogate of all other constituents in the single-pollutant model. The constituent residual model replaced the concentration of constituents within the single-pollutant model with the residuals of regression between each constituent and PM_2.5_. Furthermore, to estimate the associations of joint exposure to PM_2.5_ and its components with erythrocytosis and thrombocytosis, the quantile G-computation (QGC) method was employed additionally [34]. Briefly, the results of the QGC method include the effects of mixtures and the weight (importance) of individual exposures, and more details of these analyses are presented in Supplementary Materials. Since the effect of PM_2.5_ might be modified by population characteristics such as age, gender, and BMI, [13] stratified analyses were further conducted by age groups (<65 years and ≥65 years), gender, education levels, per capita monthly income, and BMI groups (<24 kg/m^2^ and ≥24 kg/m^2^) in the present study, and the potential effect modifications were evaluated by multiplicative interactions. In addition, according to previous studies [18,19], biological relevance, and findings of the univariate analyses, the multivariable adjustment models adjusted age, gender, marital status, education level, per capita monthly income, physical activity, smoking and drinking status, high-fat diet, adequate vegetable and fruit intake, and BMI. All data were analyzed by SPSS version 21.0 and R software version 4.2.3. The statistical significance was set at a two-tailed *p* < 0.05.

## 3. Results

### 3.1. Characteristics of the Participants

As presented in Table 1, the prevalence of erythrocytosis and thrombocytosis was 10.42% and 0.99%, respectively. Participants with both erythrocytosis and thrombocytosis tended to be younger, have inadequate vegetable and fruit intake, lower physical activity, and a higher BMI. However, the descriptions of other characteristics differed between erythrocytosis and thrombocytosis. Specifically, subjects with erythrocytosis had the following characteristics: men, married/cohabiting, high education and income levels, ever/current smoking or drinking, high-fat diet, and low PLT; while subjects suffered from thrombocytosis were more likely to be women, never smoking or drinking, and had low-fat diet, low levels of HGB and HCT. Importantly, the concentrations of PM_2.5_ and its constituents averaged over a three-year period were higher in the erythrocytosis group than the non-erythrocytosis group, as well as in the thrombocytosis group than the non-thrombocytosis group (all *p* < 0.001).

Table 2 displayed the mean, SD, median, and interquartile range (IQR) of the three-year averaged concentrations of PM_2.5_ and each constituent. The mean (SD) of PM_2.5_, BC, NH_4_^+^, NO_3_^−^, OM, SO_4_^2−^, and SOIL were 73.93 (9.67), 5.09 (0.98), 10.42 (1.35), 17.54 (2.51), 15.61 (1.88), 14.26 (1.72) and 9.75 (1.70) μg/m^3^, respectively. The median (IQR) of PM_2.5_, BC, NH_4_^+^, NO_3_^−^, OM, SO_4_^2−^, and SOIL were 77.87 (17.61), 5.49 (2.00), 10.68 (2.21), 18.89 (3.82), 15.38 (3.00), 13.86 (3.07) and 8.96 (3.56) μg/m^3^, respectively. The findings of the high positive correlations (r range from 0.63 to 0.99) between them are shown in Supplementary Figure S1.

### 3.2. Associations of PM_2.5_ and Its Constituents with Erythrocytosis and Thrombocytosis

**Figure 1:**
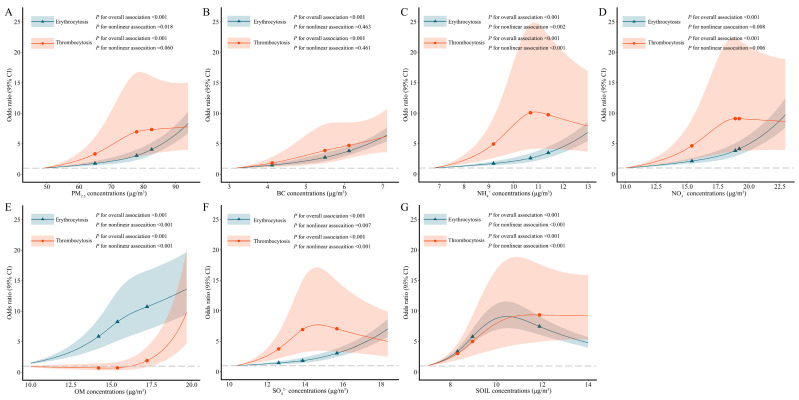
The exposure–response associations of 3-year averaged concentrations of PM_2.5_, and its constituents with erythrocytosis and thrombocytosis. Abbreviation: PM_2.5_: fine particulate matter; BC: black carbon; NH_4_^+^: ammonium; NO_3_^−^: nitrate; OM: organic matter; SO_4_^2−^: inorganic sulfate; SOIL: soil particles. Model adjusted age, gender, marital status, education level, per capita monthly income, physical activity, smoking and drinking status, high-fat diet, adequate vegetable and fruit intake, and BMI. Panel (**A**–**G**) showed the exposure–response associations of PM_2.5_, BC, NH_4_^+^, NO_3_^−^, OM, SO_4_^2−^, SOIL with erythrocytosis and thrombocytosis, respectively. Dots and triangles represented the 25th, 50th, and 75th percentiles of pollutants.

Table 3 exhibits that long-term exposure to PM_2.5_ and its six constituents were positively associated with prevalent erythrocytosis and thrombocytosis. The single-pollutant model estimated the odds ratio (*OR*) and 95% confidence interval (*CI*) of 1.049 (1.044, 1.054) for erythrocytosis and 1.040 (1.027, 1.054) for thrombocytosis, associated with 1 μg/m^3^ increase in PM_2.5_ concentrations. Moreover, the elevated risk of erythrocytosis prevalence was significantly related to all six PM_2.5_ constituents. The *OR*s and 95% *CI*s were 1.598 (1.528, 1.671) for BC, 1.407 (1.363, 1.452) for NH_4_^+^, 1.206 (1.185, 1.227) for NO_3_^−^, 1.230 (1.199, 1.261) for OM, 1.296 (1.265, 1.328) for SO_4_^2−^, and 1.196 (1.168, 1.224) for SOIL. Thrombocytosis showed similar associations with PM_2.5_ constituents, with the *OR*s and 95% *CI*s for BC, NH_4_^+^, NO_3_^−^, OM, SO_4_^2−^, and SOIL were 1.568 (1.372, 1.792), 1.220 (1.118, 1.332), 1.134 (1.080, 1.191), 1.346 (1.245, 1.456), 1.135 (1.061, 1.214), and 1.292 (1.205, 1.385), respectively. Figure 1 illustrates that most of the dose–response relationships of PM_2.5_ and its constituents with the risk of erythrocytosis and thrombocytosis were nonlinear (P for nonlinear associations < 0.05), which showed no signs of threshold.

The results of the constituent-PM_2.5_ model and the constituent residual model were consistent. NH_4_^+^, NO_3_^−^, and SO_4_^2−^ showed consistent and stronger associations with erythrocytosis. The *OR*s and 95% *CI*s were 1.317 (1.149, 1.508) and 1.152 (1.059, 1.254), 1.137 (1.058, 1.220) and 1.125 (1.044, 1.211), and 1.152 (1.059, 1.254) and 1.310 (1.202, 1.428) in the constituent-PM_2.5_ model and the constituent residual model, respectively. Additionally, each 1 μg/m^3^ increase in exposure to BC, OM, and SOIL corresponded to the elevated risk of thrombocytosis. The *OR*s and 95% *CI*s were 4.316 (2.259, 8.246), 1.680 (1.416, 1.992), and 1.232 (1.114, 1.364) in the constituent-PM_2.5_ model, and 4.053 (2.216, 7.412), 1.547 (1.316, 1.819), and 1.257 (1.134, 1.392) in the constituent residual model.

**Figure 2:**
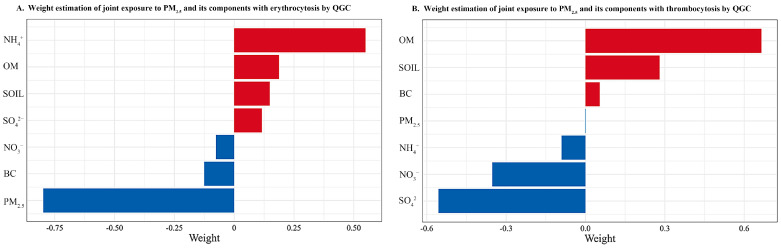
Weight estimations of joint exposure to PM_2.5_ and its components with erythrocytosis and thrombocytosis. Abbreviation: PM_2.5_: fine particulate matter; BC: black carbon; NH_4_^+^: ammonium; NO_3_^−^: nitrate; OM: organic matter; SO_4_^2−^: inorganic sulfate; SOIL: soil particles. Model adjusted age, gender, marital status, education level, per capita monthly income, physical activity, smoking and drinking status, high-fat diet, adequate vegetable and fruit intake, and BMI.

Figure 2 shows the results of the QGC method. Among PM_2.5_ and its constituents, NH_4_^+^ had the largest estimated weight for erythrocytosis, indicating its significant contribution to this disease. On the other hand, OM had the largest estimated weight for thrombocytosis, suggesting its importance in the development of this condition. The joint exposure to PM_2.5_ and its constituents was positively associated with erythrocytosis and thrombocytosis, and the *ORs* and 95% *CIs* were 1.542 (1.352, 1.761) for erythrocytosis and 1.522 (1.458, 1.590) for thrombocytosis.

### 3.3. Stratified Analyses

Supplementary Figure S2 shows the finding of stratified analyses examining the relationships of PM_2.5_ and its constituents with erythrocytosis and thrombocytosis. The results showed that the magnitude of positive associations of PM_2.5_ and its constituents with erythrocytosis were stronger among women, individuals with a low education level, and those with a low BMI. These characteristics had a significant modifying effect on the relationships of PM_2.5_ and its constituents with erythrocytosis (P for interaction < 0.05). However, no such significant modifying effect of the above population characteristic was observed on the correlations of PM_2.5_ and its constituents with thrombocytosis.

## 4. Discussion

To the best of our knowledge, this study is the first to examine the associations of PM_2.5_ with prevalent erythrocytosis and thrombocytosis. The results of this study indicate positive associations of PM_2.5_ and its six constituents with prevalent erythrocytosis and thrombocytosis. Importantly, this study suggests that NH_4_^+^ may be responsible for the association between PM_2.5_ and erythrocytosis, and thrombocytosis may be attributed to OM as the responsible component.

While evidence on the associations of PM_2.5_ with prevalent erythrocytosis and thrombocytosis is lacking, several investigations have investigated the effects of PM_2.5_ on erythrocyte and platelet parameters, yielding different and even contradictory results. For instance, in a study conducted in Denmark, the research found that women who experienced a 10 μg/m^3^ increase in personal PM_2.5_ exposure showed a 2.6% increase in HGB and a 2.3% increase in RBC based on repeated assessments of PM_2.5_ exposure [16]. Another study conducted in Chinese cities, using both panel and cross-sectional designs, found that PM_2.5_ had a positive association with PLT but a negative association with RBC [13]. Researchers in the Korean Children’s Environmental Health Study observed a negative correlation between indoor PM_2.5_ and HGB and HCT levels [14]. Furthermore, a recent investigation in Beijing reported that elevated PM_2.5_ levels were associated with a decrease in PLT [15]. These inconsistent associations may be partly attributed to differences in study design and population characteristics, such as age, gender, BMI, and psychosocial stress [13].

Numerous studies have suggested that the toxicity of PM_2.5_ depends on its chemical composition, with each constituent having distinct impacts [18,32,35,36]. For example, previous research has shown that BC is strongly correlated with glucose metabolism [18], while OC, EC, NH_4_^+^, NO_3_^−^, and SO_4_^2−^ are more strongly associated with inflammatory or coagulation markers [32]. Consistent with these findings, both the constituent-PM_2.5_ model and the constituent-residual model in the current study demonstrated that NH_4_+, NO_3_^−^, and SO_4_^2−^ were strongly associated with erythrocytosis, while BC, OM, and SOIL had robust associations with thrombocytosis. Moreover, the prevalence of erythrocytosis in this study was much higher than in previous research, which may be attributed to significantly higher PM_2.5_ concentrations in rural areas of China compared to European countries [37].

Interestingly, in the current study, NH_4_^+^ (a component of secondary inorganic aerosols including NH_4_^+^, NO_3_^−^, and SO_4_^2−^) was found to be responsible for the association between PM_2.5_ and erythrocytosis. These aerosols are predominantly formed through chemical reactions of gaseous precursors emitted into the atmosphere, such as SO_2_, NO_x_, and NH_3_^+^ [38]. Recent research has indicated a more significant decrease in primary PM_2.5_ pollutants compared to secondary PM_2.5_ pollutants as a result of China’s clean air plan [39]. As the proportion of secondary PM_2.5_ increases, it may have implications for various diseases, including erythrocytosis. On the other hand, this study revealed that OM was responsible for the association between PM_2.5_ and thrombocytosis. These findings underscore the importance of addressing the energy structure in rural areas, where polluting fuels, the primary source of OM, are commonly used by rural residents for cooking and heating purposes [40,41,42].

The underlying mechanisms linking long-term exposure to PM_2.5_ with prevalent erythrocytosis and thrombocytosis are unclear. However, there is evidence to suggest that PM_2.5_ exposure can increase the level of reticulocytes in peripheral blood. This, in turn, may stimulate the release of erythropoietin from the bone marrow, a hormone that promotes the production of RBCs [43]. Additionally, exposure to transition metals, such as cobalt and manganese, bound to PM_2.5_ has been found to directly stimulate erythropoiesis. These metals have also been associated with the induction effect of hypoxia-inducible factor 1 on erythropoietin [5,43]. The biological mechanism underlying the relationship between PM_2.5_ and thrombocytosis may involve systemic inflammation, oxidative stress, and impaired endothelial cells. PM_2.5_ exposure can trigger systemic inflammation through the Toll-like receptor (TLR) 4 pathway, which has been shown to modulate the production and activation of platelets [44,45]. In addition, air pollutants have the ability to induce platelet aggregation in injured vascular endothelium and stimulate megakaryocytes to produce platelets [46]. However, the exact reason why certain constituents are more strongly associated with erythrocytosis or thrombocytosis remains unclear. Further investigations are required to determine the potentially harmful effects of various components implicated in erythrocytosis or thrombocytosis.

This study provides significant implications for public health strategies. Firstly, it highlights the positive associations of PM_2.5_ and its constituents with prevalent erythrocytosis and thrombocytosis, with each constituent being strongly associated with one of the outcomes. Indeed, rural residents are more vulnerable to PM_2.5_ exposure due to limited access to medical resources and higher levels of pollution compared to urban areas [47,48]. Therefore, reducing emissions of PM_2.5_ from all sources in rural regions is crucial and can lead to significant health benefits. Second, considering the variations in the sources and relative contributions of PM_2.5_ constituents, along with the limited financial and health resources in rural areas, targeted interventions focusing on specific pollutant sources can be more effective in reducing adverse health outcomes. By identifying the key contributors to PM_2.5_ pollution in specific rural regions, resources can be allocated more efficiently to address those specific sources. This approach can lead to better air quality and improved health outcomes for the residents. To sum up, this study highlights the urgent need to address PM_2.5_ pollution, particularly in rural areas, and provides insights into the potential strategies for reducing its adverse health effects. By implementing comprehensive measures to reduce PM_2.5_ emissions and adopting targeted interventions, public health can be significantly improved, benefiting the overall well-being of the population.

This study has several limitations that should be acknowledged. Firstly, the cross-section design used in this study limits the ability to establish causation, highlighting the need for further prospective studies to confirm the findings. Meanwhile, this study employed an ecological epidemiological design, which estimated community-level exposure using models and then extrapolated it to individuals based on their residential locations. Individual exposure levels can vary based on factors such as wind speed, humidity, and the accuracy of the models used for estimation. Secondly, due to the lack of a perfectly comprehensive monitoring system for PM_2.5_ components in China, the individual exposures evaluated using the Geos-Chem model were not calibrated in this study. However, a previous study has confirmed the accuracy of the estimated PM_2.5_ concentrations with an R^2^ value of 0.81 [31], and these estimates have been successfully applied in the Chinese population [18,49]. Finally, while many important confounding factors were adjusted for, there may still be unmeasured and unknown confounders that could influence the results.

## 5. Conclusions

In summary, this study found a positive association between long-term exposure to PM_2.5_ and its constituents with prevalent erythrocytosis and thrombocytosis. The study suggests that NH_4_^+^ may be responsible for the association between PM_2.5_ and erythrocytosis, while the association between PM_2.5_ and thrombocytosis may be attributed to OM as the responsible component. These findings highlight the differences in the relative contributions of constituents and underscore the urgent need for PM_2.5_ pollution control in rural areas.

## Figures and Tables

**Table 1:** Basic characteristics of study participants.

Variables	Erythrocytosis	Non-Erythrocytosis	*p*-Value	Thrombocytosis	Non-Thrombocytosis	*p*-Value
	(n = 3499)	(n = 30,086)		(n = 333)	(n = 33,252)	
Age (year), mean ± SD	52.68 ± 13.06	55.58 ± 12.15	<0.001	52.54 ± 12.54	55.31 ± 12.28	<0.001
Gender, n (%)			<0.001			<0.001
Men	2926 (83.62)	10,648 (35.39)		56 (16.82)	13,518 (40.65)	
Women	573 (16.38)	19,438 (64.61)		277 (83.18)	19,734 (59.35)	
Marital status, n (%)			0.021			0.897
Married/cohabiting	3194 (91.28)	27,093 (90.05)		301 (90.39)	29,986 (90.18)	
Unmarried/divorced/widowed	305 (8.72)	2993 (9.95)		32 (9.61)	3266 (9.82)	
Education levels, n (%)			<0.001			0.172
Primary school or below	1048 (29.95)	13,634 (45.32)		149 (44.74)	14,533 (43.71)	
Junior high school	1623 (46.38)	11,798 (39.21)		142 (42.64)	13,279 (39.93)	
Senior high school or above	828 (23.67)	4654 (15.47)		42 (12.62)	5440 (16.36)	
Per capita monthly income, n (%)			<0.001			0.467
<500 RMB	1111 (31.75)	10,772 (35.80)		122 (36.64)	11,761 (35.37)	
500–999 RMB	1143 (32.67)	9789 (32.54)		98 (29.43)	10,834 (32.58)	
≥1000 RMB	1245 (35.58)	9525 (31.66)		113 (33.93)	10,657 (32.05)	
Smoking status, n (%)			<0.001			<0.001
Never	1384 (39.56)	22,851 (75.95)		287 (86.19)	23,948 (72.02)	
Ever	513 (14.66)	2151 (7.15)		20 (6.01)	2644 (7.95)	
Current	1602 (45.78)	5084 (16.90)		26 (7.80)	6660 (20.03)	
Drinking status, n (%)			<0.001			<0.001
Never	1756 (50.18)	24,183 (80.38)		301 (90.39)	25,638 (77.1)	
Ever	276 (7.89)	1310 (4.35)		5 (1.50)	1581 (4.75)	
Current	1467 (41.93)	4593 (15.27)		27 (8.11)	6033 (18.14)	
Physical activity, n (%)			<0.001			0.013
Low	1353 (38.67)	9368 (31.14)		129 (38.74)	10,592 (31.85)	
Moderate	1065 (30.44)	11,611 (38.59)		104 (31.23)	12,572 (37.81)	
High	1081 (30.89)	9107 (30.27)		100 (30.03)	10,088 (30.34)	
High fat diet, n (%)	898 (25.66)	5388 (17.91)	<0.001	46 (13.81)	6240 (18.77)	0.021
Adequate vegetable and fruit intake, n (%)	1363 (38.95)	13,716 (45.59)	<0.001	129 (38.74)	14,950 (44.96)	0.023
BMI (kg/m^2^, mean ± SD)	25.44 ± 3.47	24.71 ± 3.56	<0.001	25.79 ± 3.43	24.78 ± 3.56	<0.001
HGB (g/L, mean ± SD)	166.86 ± 16.92	135.17 ± 14.62	<0.001	119.90 ± 24.77	138.66 ± 17.57	<0.001
HCT (%, mean ± SD)	52.00 ± 4.94	42.10 ± 3.89	<0.001	39.03 ± 6.47	43.17 ± 4.99	<0.001
PLT (10^9^/L, mean ± SD)	206.62 ± 64.60	216.15 ± 71.75	<0.001	437.78 ± 28.40	212.92 ± 67.80	<0.001
PM_2.5_ (μg/m^3^, mean ± SD)	77.69 ± 8.99	73.50 ± 9.66	<0.001	77.75 ± 8.77	73.90 ± 9.68	<0.001
Constituents						
BC (μg/m^3^, mean ± SD)	5.48 ± 0.92	5.05 ± 0.98	<0.001	5.52 ± 0.89	5.09 ± 0.98	<0.001
NH_4_^+^ (μg/m^3^, mean ± SD)	10.95 ± 1.31	10.36 ± 1.35	<0.001	10.85 ± 1.22	10.42 ± 1.36	<0.001
NO_3_^−^ (μg/m^3^, mean ± SD)	18.49 ± 2.37	17.43 ± 2.50	<0.001	18.40 ± 2.28	17.54 ± 2.51	<0.001
OM (μg/m^3^, mean ± SD)	16.22 ± 1.62	15.55 ± 1.90	<0.001	16.49 ± 1.81	15.61 ± 1.88	<0.001
SO_4_^2−^ (μg/m^3^, mean ± SD)	14.95 ± 1.70	14.19 ± 1.71	<0.001	14.74 ± 1.57	14.26 ± 1.73	<0.001
SOIL (μg/m^3^, mean ± SD)	10.31 ± 1.67	9.69 ± 1.69	<0.001	10.57 ± 1.63	9.75 ± 1.70	<0.001

Student’s *t*-test and chi-square test were performed to compare the differences of continuous variables and categorical variables, respectively. Abbreviation: SD, standard deviation; RMB, Renminbi; BMI, body mass index; PM_2.5_: fine particulate matter; BC: black carbon; NH_4_^+^: ammonium; NO_3_^−^: nitrate; OM: organic matter; SO_4_^2−^: inorganic sulfate; SOIL: soil particles.

**Table 2:** Summary distributions of PM_2.5_ and its constituents during the study.

Variables	Mean	SD	Minimum	Median	Maximum	IQR
PM_2.5_ (μg/m^3^)	73.93	9.67	48.49	77.87	94.09	17.61
Constituents						
BC (μg/m^3^)	5.09	0.98	3.11	5.49	7.11	2.00
NH_4_^+^ (μg/m^3^)	10.42	1.35	6.74	10.68	13.65	2.21
NO_3_^−^ (μg/m^3^)	17.54	2.51	9.97	18.89	23.41	3.82
OM (μg/m^3^)	15.61	1.88	8.74	15.38	19.74	3.00
SO_4_^2−^ (μg/m^3^)	14.26	1.72	10.33	13.86	18.36	3.07
SOIL (μg/m^3^)	9.75	1.70	6.94	8.96	14.03	3.56

Abbreviation: SD, standard deviation; IQR, interquartile range; PM_2.5_: fine particulate matter; BC: black carbon; NH_4_^+^: ammonium; NO_3_^−^: nitrate; OM: organic matter; SO_4_^2−^: inorganic sulfate; SOIL: soil particles.

**Table 3:** The associations of single ambient PM_2.5_ and its constituents (μg/m^3^) with erythrocytosis and thrombocytosis.

Variables	Single-Pollutant Model ^a^	Constituent-PM_2.5_ Model ^b^	Constituent Residual Model ^c^
Erythrocytosis			
PM_2.5_	1.049 (1.044, 1.054)		
Constituents			
BC	1.598 (1.528, 1.671)	1.072 (0.852, 1.348)	1.054 (0.839, 1.325)
NH_4_^+^	1.407 (1.363, 1.452)	1.317 (1.149, 1.508)	1.521 (1.318, 1.756)
NO_3_^−^	1.206 (1.185, 1.227)	1.137 (1.058, 1.220)	1.125 (1.044, 1.211)
OM	1.230 (1.199, 1.261)	0.818 (0.771, 0.869)	0.777 (0.736, 0.821)
SO_4_^2−^	1.296 (1.265, 1.328)	1.152 (1.059, 1.254)	1.310 (1.202, 1.428)
SOIL	1.196 (1.168, 1.224)	0.979 (0.946, 1.013)	0.980 (0.947, 1.015)
Thrombocytosis			
PM_2.5_	1.040 (1.027, 1.054)		
Constituents			
BC	1.568 (1.372, 1.792)	4.316 (2.259, 8.246)	4.053 (2.216, 7.412)
NH_4_^+^	1.220 (1.118, 1.332)	0.228 (0.141, 0.369)	0.158 (0.092, 0.272)
NO_3_^−^	1.134 (1.080, 1.191)	0.729 (0.594, 0.895)	0.692 (0.559, 0.856)
OM	1.346 (1.245, 1.456)	1.680 (1.416, 1.992)	1.547 (1.316, 1.819)
SO_4_^2−^	1.135 (1.061, 1.214)	0.433 (0.346, 0.542)	0.434 (0.347, 0.541)
SOIL	1.292 (1.205, 1.385)	1.232 (1.114, 1.364)	1.257 (1.134, 1.392)

Abbreviation: PM_2.5_: fine particulate matter; BC: black carbon; NH_4_^+^: ammonium; NO_3_^−^: nitrate; OM: organic matter; SO_4_^2−^: inorganic sulfate; SOIL: soil particles. All models adjusted age, gender, marital status, education level, per capita monthly income, physical activity, smoking and drinking status, high-fat diet, adequate vegetable and fruit intake, and BMI. ^a^ Single-pollutant model regarded the concentration of PM_2.5_ mass and constituents as independent variable and with adjustment of covariates. ^b^ Constituent-PM_2.5_ model further adjusted PM_2.5_ on the basis of constituent concentration model. ^c^ Constituent-residual model replaced the concentration of constituents in the single-pollutant model with the residuals of regression between each constituent and PM_2.5_.

## Data Availability

The data analyzed during the current study are available from the corresponding author upon reasonable request.

## Supplementary Materials

The following supporting information can be downloaded at: https://www.mdpi.com/article/10.3390/toxics11110885/s1, Figure S1: Correlation between 3-year averaged concentrations of ambient PM_2.5_ and its constituents; Figure S2: The associations of 3-year averaged concentrations of PM_2.5_ and its constituents with erythrocytosis and thrombocytosis across general characteristics.
