# Supplementary material for: Associations of Long-Term Exposure to PM2.5 and Its Constituents with Erythrocytosis and Thrombocytosis in Rural Populations
Source: Toxics. 2023 Oct 27;11(11):885. doi: 10.3390/toxics11110885 (PMC10674504; doi:10.3390/toxics11110885)
Supplement: Supplementary file 1 [file toxics-11-00885-s001.zip › toxics-2646518-supplementary.pdf]

Supplementary appendix

This appendix formed part of the original submission and has been peer reviewed. We post it as supplied by the authors.

Supplement to: Associations of long-term exposure to $PM_{2.5}$ and its constituents with erythrocytosis and thrombocytosis in rural populations

Authors:

Yiquan Zheng[a], Yaling He[b], Ning Kang[a], Caiyun Zhang[a], Wei Liao[a], Yinghao Yuchi[a], Xiaotian Liu[a], Jian Hou[a], Zhenxing Mao[a], Wenqian Huo[a], Kai zhang[c], Hezhong Tian[d], Hualiang Lin[a,e], Chongjian Wang[a*]

Authors affiliations:

[a] Department of Epidemiology and Biostatistics, College of Public Health, Zhengzhou University, Zhengzhou, Henan, *PR* China.

[b] Department of Occupational and Environmental Health, Ministry of Education Key Laboratory of Environment and Health, and State Key Laboratory of Environmental Health (Incubating), School of Public Health, Tongji Medical College, Huazhong University of Science and Technology, Wuhan, Hubei, China.

[c] Department of Environmental Health Sciences, School of Public Health, University at Albany, State University of New York, Albany, NY USA.

[d] State Key Joint Laboratory of Environmental Simulation & Pollution Control, School of Environment, Beijing Normal University, Beijing, *PR* China.

[e] Department of Epidemiology, School of Public Health, Sun Yat-sen University, Guangzhou, Guangdong, *PR* China.

*** Correspondence author**

Prof. Chongjian Wang

Department of Epidemiology and Biostatistics

College of Public Health, Zhengzhou University

100 Kexue Avenue, Zhengzhou, 450001, Henan, *PR* China

Phone : +86 371 67781452 ;

Fax : +86 371 67781919

E-mail : tjwcj2008@zzu.edu.cn

Expand methods

Exposure assessment of $PM_{2.5}$ constituents

As reported previous [1], the PM2.5 constituents in this study was simulated by GEOS-Chem Chemical transport models (GEOS-Chem CTM, version 11−01; http://www.geos-chem. org). The GEOS-Chem model simulates the temporal and three-dimensional spatial distributions of various aerosol components and gases using assimilated meteorology and emission inventories as major inputs. This simulation includes carbonaceous aerosols, mineral dust aerosols, secondary organic aerosols, sea salt aerosols, and sulfate aerosols. The detailed stimulation of GEOS-Chem was complex and described in Aerosols section of overview [2]. GEOS-Chem carbonaceous aerosols include BC, organic carbon (OC), and secondary organic aerosols (SOA), and the OM was calculated as the sum of model OC and SOA [3]. The simulation of SOIL in GEOS-Chem is sampled according to the site observations, which are based on the sum of the soil-derived elements (Al, Si, K, Ca, Ti, and Fe) and their normal oxides [4-5]. The SS simulation in the GEOS-Chem model was first implemented by Alexander et al. using the source function described by Monahan et al. [6]. In the current simulation (GEOS-Chem CTM, version 11−01) of SS, the updated source function was derived by Jaegle et al. [6]. Moreover, GEOS-Chem is complex and includes the efforts of various experts, and the detailed links could be found in elsewhere [1-2].

Association of $PM_{2.5}$ constituents with erythrocytosis and thrombocytosis

Multiple logistic regression was employed to assess the associations of PM2.5 mass and its constituents with erythrocytosis and thrombocytosis. To specify more responsible constituents, the constituent-PM2.5 model and constituent residual model were further used in addition to the single-pollutant model. The main model can be presented by the following formulas [7]:

$g(\mu) = \beta 0 + \beta 1(\text{constituent or PM2.5}) + [\gamma' X]$ (a)

$g(\mu) = \beta 0 + \beta 1(\text{constituent}) + \beta 2(\text{PM2.5}) + [\gamma' X]$ (b)

$g(\mu) = \beta 0 + \beta 1(\text{residual}) + [\gamma' X]$ (c)

in which formula (a) was single-pollutant model, formula (b) was constituent-PM2.5 model and formula (c) was constituent residual model. In each formula, the μ was health outcome; the g() was the link function (logit for erythrocytosis and thrombocytosis); the β0 represented the intercept; β1 represented the coefficient of exposure; residual in formula (c) represented the

residuals of linear regression (consistent = $\beta0 + \beta(PM2.5)$); [γ'X] was matrix of other covariates (age, gender, marital status, education level, per capita monthly income, physical activity, smoking and drinking status, high-fat diet, adequate vegetable and fruit intake, and BMI).

Quantile G-computation

Quantile G-computation is a new method for analyzing environmental mixtures which integrates G-computation based on Weighted Quantile Sum (WQS) regression. [8] G-computation is a commonly used method in causal inference. Compared with WQS, quantile G-computation estimates the overall mixture effect with the same procedure but estimates the parameters of a marginal structural model rather than a standard regression in WQS. This way, under common assumptions in causal inference such as exchangeability, causal consistency, positivity, no interference, and correct model specification, this model will also improve the causal interpretation of the overall effect. In addition, the procedure also allegedly overcomes the assumption of uni-direction in WQS, which also means that quantile G-computation can estimate both positive and negative weights at the same time.

Reference

1. Li, C.; Martin, R.V.; van Donkelaar, A.; Boys, B.L.; Hammer, M.S.; Xu, J.W.; Marais, E.A.; Reff, A.; Strum, M.; Ridley, D.A.; et al. Trends in Chemical Composition of Global and Regional Population-Weighted Fine Particulate Matter Estimated for 25 Years. *Environ. Sci. Technol.* **2017**, *51*, 11185–11195. https://doi.org/10.1021/acs.est.7b02530.
2. http://wiki.seas.harvard.edu/geos-chem/index.php/GEOS-Chem_overview (accessed on 23 March 2023)
3. http://wiki.seas.harvard.edu/geos-chem/index.php/Carbonaceous_aerosols (accessed on 24 March 2023)
4. http://wiki.seas.harvard.edu/geos-chem/index.php/Mineral_dust_aerosols (accessed on 24 March 2023)
5. Zhang, L., Kok, J F.; Henze, D; Li, Q; Zhao, C. Improving simulations of fine dust surface concentrations over the western United States by optimizing the particle size distribution. *Geophysical Research Letters*. **2013**, *40*, 3270-3275. https://doi.org/10.1002/grl.50591
6. http://wiki.seas.harvard.edu/geos-chem/index.php/Sea_salt_aerosols (accessed on 24 March 2023)
7. Mostofsky, E.; Schwartz, J.; Coull, B.A.; Koutrakis, P.; Wellenius, G.A.; Suh, H.H.; Gold, D.R. and Mittleman, M.A. Modeling the association between particle constituents of air pollution and health outcomes. *Am J Epidemiol*. **2012**, *176*, 317-26. https://doi.org/10.1093/aje/kws018.
8. Keil, A.P.; Buckley, J.P.; O'Brien, K.M.; Ferguson, K.K.; Zhao, S. and White, A.J. A Quantile-Based g-Computation Approach to Addressing the Effects of Exposure Mixtures. *Environ Health Perspect*. **2020**, *128*, 47004. https://doi.org/10.1289/ehp5838.

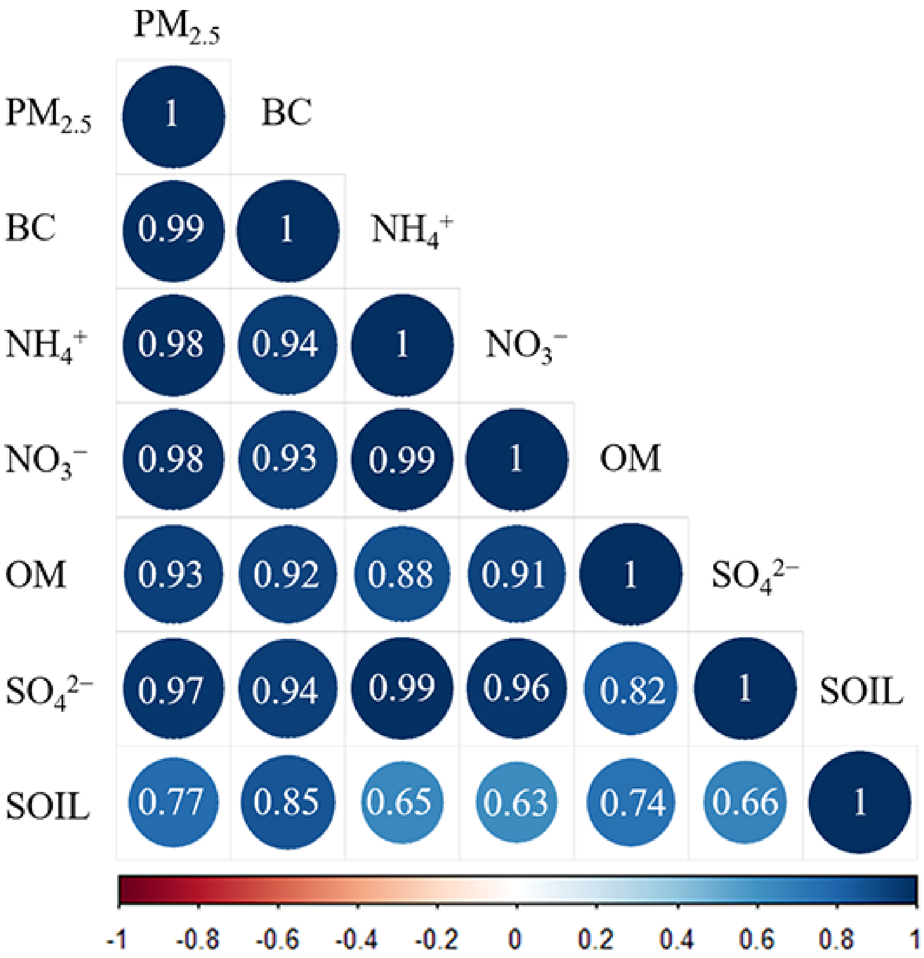

Supplementary Figure S1. Correlation between 3-year averaged concentrations of ambient $PM_{2.5}$ and its constituents (Abbreviation: $PM_{2.5}$: fine particulate matter; BC: black carbon; NH_4^+: ammonium; NO_3^-: nitrate; OM: organic matter; SO_4^{2-}: inorganic sulfate; SOIL: soil particles).

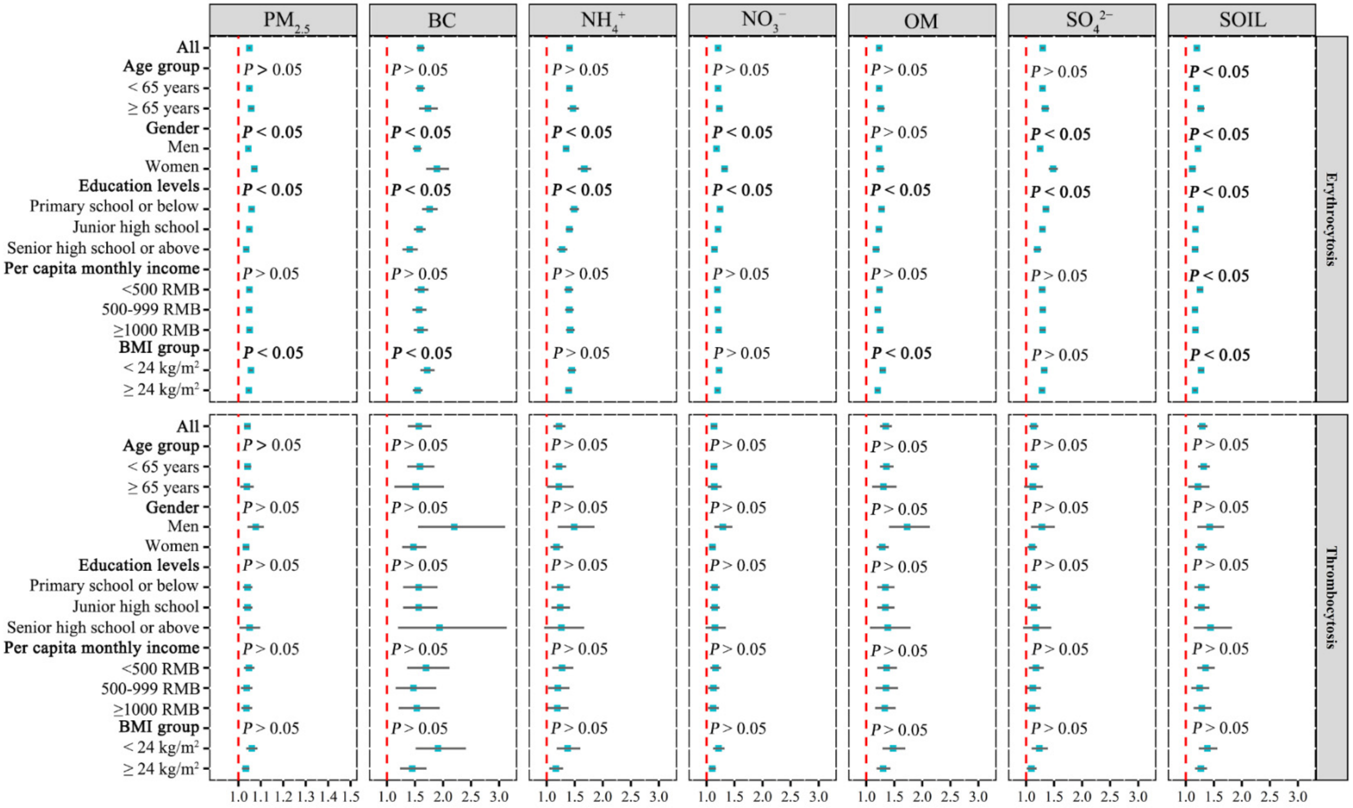

Supplementary Figure S2. The associations of 3-year averaged concentrations of $PM_{2.5}$ and its constituents with erythrocytosis and thrombocytosis across general characteristics (Abbreviation: $PM_{2.5}$: fine particulate matter; BC: black carbon; NH_4^+: ammonium; NO_3^-: nitrate; OM: organic matter; SO_4^{2-}: inorganic sulfate; SOIL: soil particles. Model adjusted age, gender, marital status, education level, per capita monthly income, physical activity, smoking and drinking status, high-fat diet, adequate vegetable and fruit intake, and BMI).
